# Supplementary figures and images for: An intact S-layer is advantageous to Clostridioides difficile within the host
Source: PLoS Pathog. 2023 Jun 29;19(6):e1011015. doi: 10.1371/journal.ppat.1011015 (PMC10310040; doi:10.1371/journal.ppat.1011015)

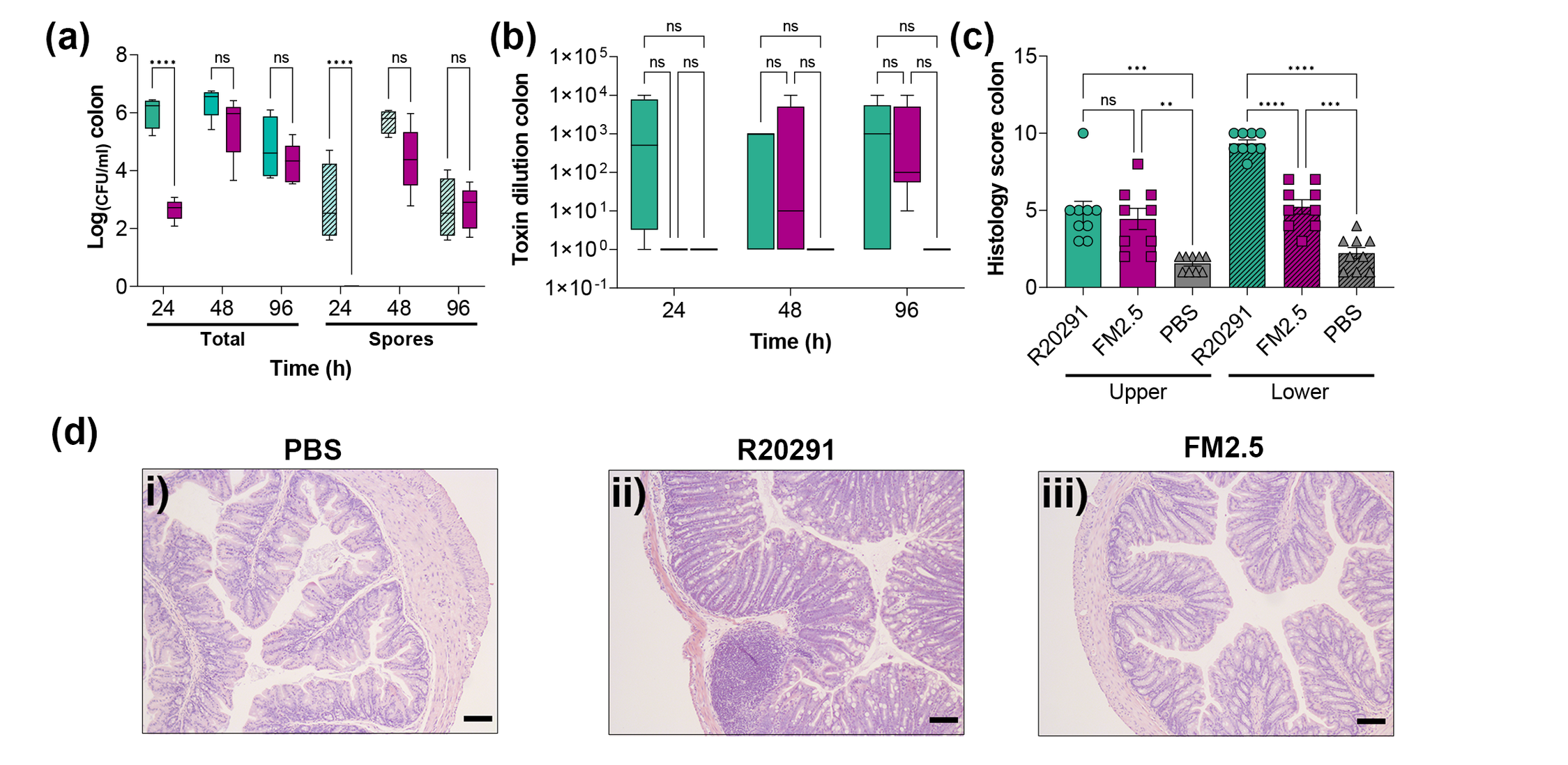

Supplement: S1 Fig — Female C57/Bl6 mice were challenged with spores of R20291 (green) or FM2.5 (purple), or mock infected with sterile PBS (grey). (a) CFU ml-1 of total (clear fill) bacterial recovery or spores (fill pattern) in colonic contents at 24, 48 and 96 hpi (n = 5 at each time point except R20291 at 24 hpi; n = 4). (b) Toxin activity within colonic content at 24, 48 and 96 hpi; through challenge of Vero cells in vitro (n = 5 at each time point). Results displayed indicate the reciprocal of lowest dilution at which toxin activity could be measured. (c) Histological scoring of sections of the upper (clear fill) and lower (fill pattern) colon sections from mice challenged with R20291, FM2.5 and PBS treated animals. Results displayed are the mean ± SEM of assessment of at least three regions of tissue from at least three individual animals. (d) Histopathological sections representing caecal (i, ii and iii) sections following challenge with PBS (i); R20291 (ii); or FM2.5 (iii). Scale bars represent 100 μm. Statistical tests were conducted using GraphPad Prism software v.12. Statistical significance is indicated: ns—not significant; *p < 0.05; **p < 0.01; and ***p < 0.001. (TIF) [file ppat.1011015.s001.tif]

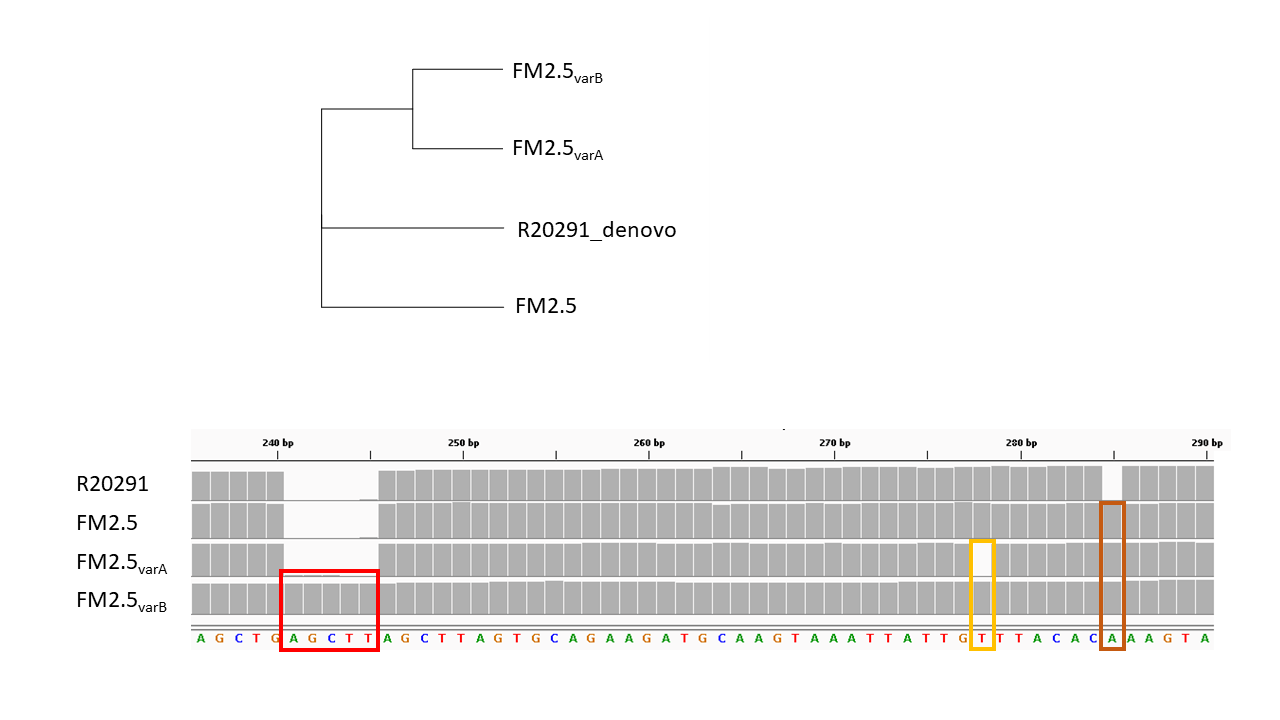

Supplement: S2 Fig — Chromosomal DNA was recovered from each strain and the raw data was aligned to the reference genome (NC_013316.1). (a) The variants were identified as closely related to both FM2.5 and R20291 using whole genomic sequencing to generate phylogenetic trees based on identification of polymorphisms identified during comparative analysis of sequences. Mutations were only included with a read depth of > 50 and which was present in at least 50% of reads. (b) Evaluation of the slpA sequence from all four strains from genomic data confirmed mutations were limited to the regions highlighted above. Mutations originally identified by PCR analysis were confirmed by short read genomic sequencing of individual strains. Insertions of the previously identified additional A in FM2.5, FM2.5varA, FM2.5varB are highlighted within the purple box, deletion of T in FM2.5varA by the dark blue box and insertion of CTTAG in FM2.5varB in red. Grey indicates the depth of sequence read coverage. (TIF) [file ppat.1011015.s002.tif]

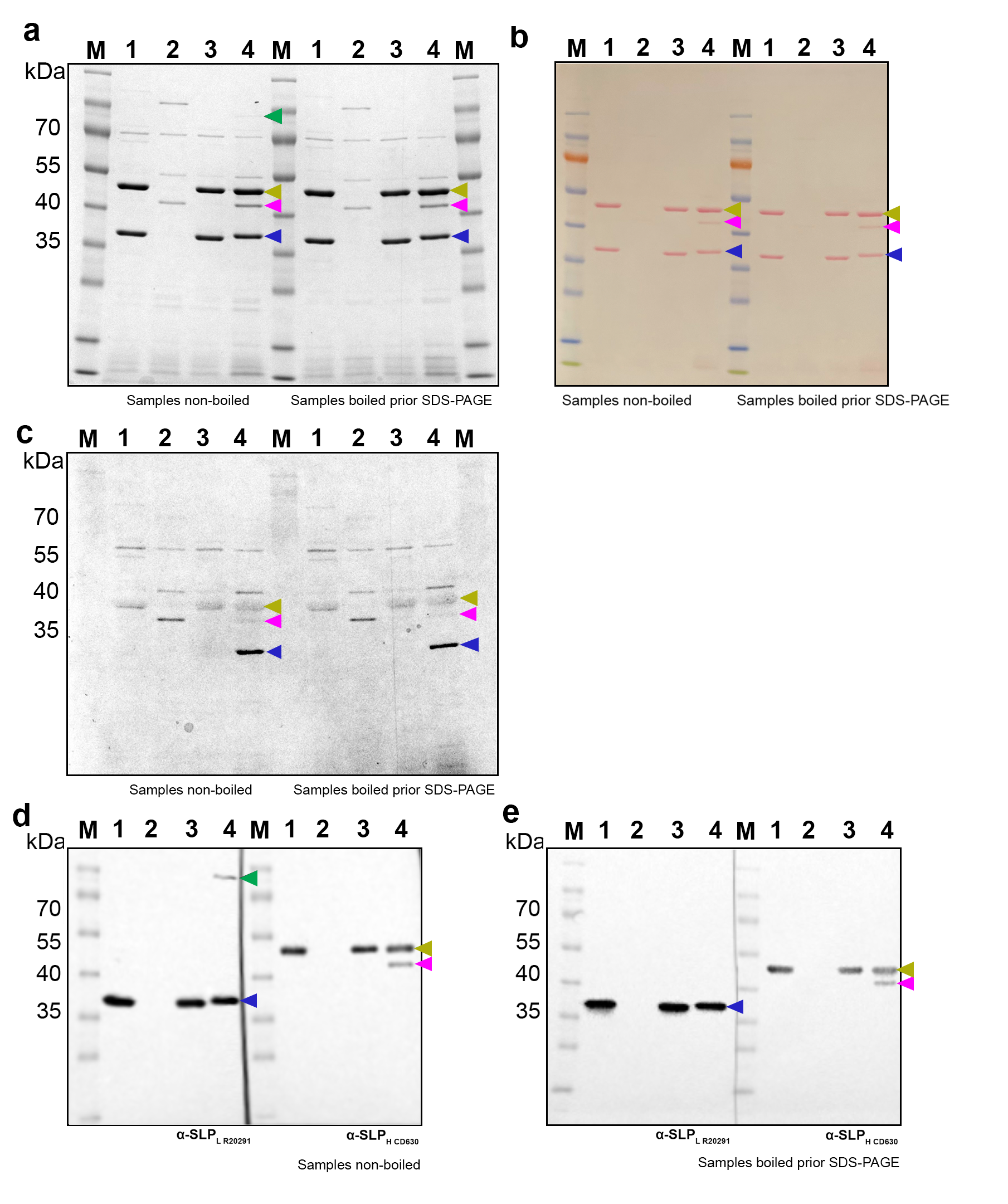

Supplement: S3 Fig — (a) SDS-PAGE of S-layer extracts, non-boiled and boiled prior to resolving on a polyacrylamide gel. Molecular weight marker (M, PageRuler Prestained Protein Ladder, ThermoScientific); R20291 (1); FM2.5 (2); FM2.5varA (3); FM2.5varB (4). For Fig 2d, the SDS-PAGE gel was cropped to show only heated samples. (b) To assess efficiency of electrotransfer, the nitrocellulose membrane was stained with PonceauS (Sigma) and (c) polyacrylamide gel post-transfer was stained with Coomassie. The Western immunoblot analysis was performed comparing (d) non-boiled and (e) boiled S-layer extracts. For Fig 2e, the blots were cropped to allow focus on the heated samples only. Throughout all images, the SLPL is indicated with blue arrowhead and SLPH is indicated with yellow arrowhead. The additional band, detected with anti-SLPH CD630 antibodies and highlighted with magenta arrowhead, corresponds to SLPH missing interacting domain HID [24]. The high molecular weight band detected with anti-SLPL R20291, indicated with green arrowhead, could correspond to an apparent SDS-resistant oligomers of SLPL or non-specific binding of the antibody to CwpV, a phase variable protein. (TIF) [file ppat.1011015.s003.tif]

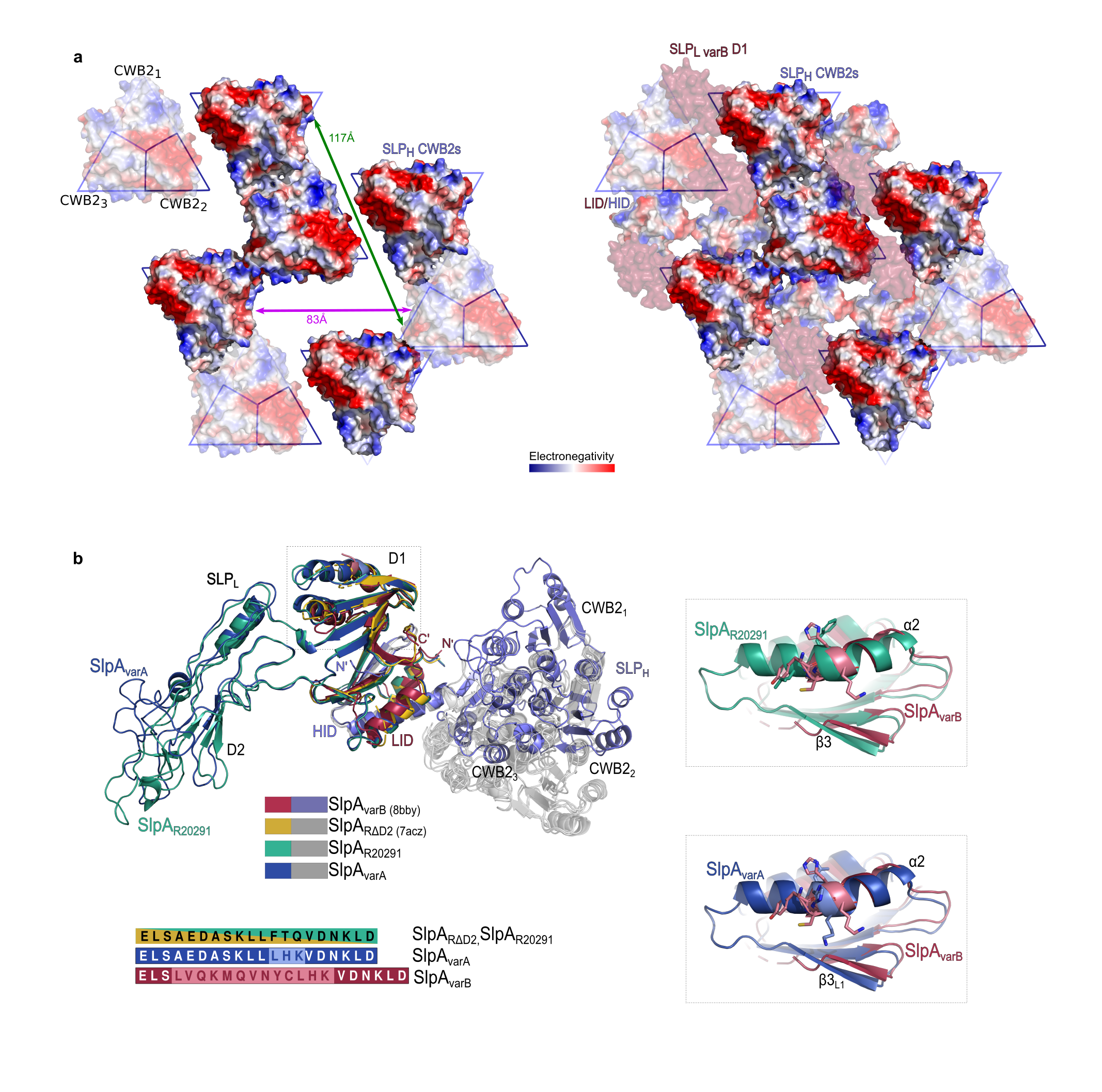

Supplement: S4 Fig — (a) Tiling of SLPH CWB2 motifs is stabilised by interacting domains. Poisson-Boltzmann electrostatic potential calculated for SlpAvarB SLPH, represented as a charge distribution (positive—blue; negative—red) on the surface representation of SLPH array. Interacting surfaces between molecules 1–2, defined by pseudo-symmetry related CWB23-CWB21, and between molecules 1–3, defined symmetry-related CWB23 triangular prism faces, are labelled. The cavity between symmetry-related CWB21-CWB22 surfaces, (117 Å, green arrow, left) is partially occluded by SLPL D1 (crimson) and by the insertion of the LID/HID domains (electrostatic potential surface representation), as shown on the right panel. A long cavity of ~83 Å at the CWB22 vertices represented by purple arrow (left) is also occluded by LID/HID domains and interacting SLPL molecules (bottom). Neighbouring CWB21-CWB23 triangular prism faces form the interaction surface, while neighbouring CWB23 vertices complete the SLPH tiling. Insertion of the interacting domains bridges neighbouring SLPH tiles. Interactions across the lattice are maintained via complementary charged interfaces. (b) Predicted structural models for SlpAR20291 (SLPL—green, SLPH—grey) and SlpAvarA (SLPL—dark blue, SLPH—grey) show the same overall structure of both SLPs as the experimental structural model for SlpAvarB (SLPL—gold, SLPH—slate blue) and SlpARΔD2 (SLPL—gold, SLPH—grey). Left: Cartoon representation of the complex, as seen from the environmental side, with all models superimposed on SLPL from SlpAvarB. The different orientation of SLPL and interaction domains observed in SlpAvarB is evidenced by the change in position of SLPH in this structure when compared to SlpARΔD2 and the predicted models, calculated based on SlpAR7404 (PDB ID: 7acx). Right: Zoom view comparing D1 region of SlpAvarB (crimson) with SlpAR20291 (top right, green) and SlpAvarA (bottom right, dark blue). Sequence of α2L in the different variants, with paler colours indicating di [file ppat.1011015.s004.tiff]

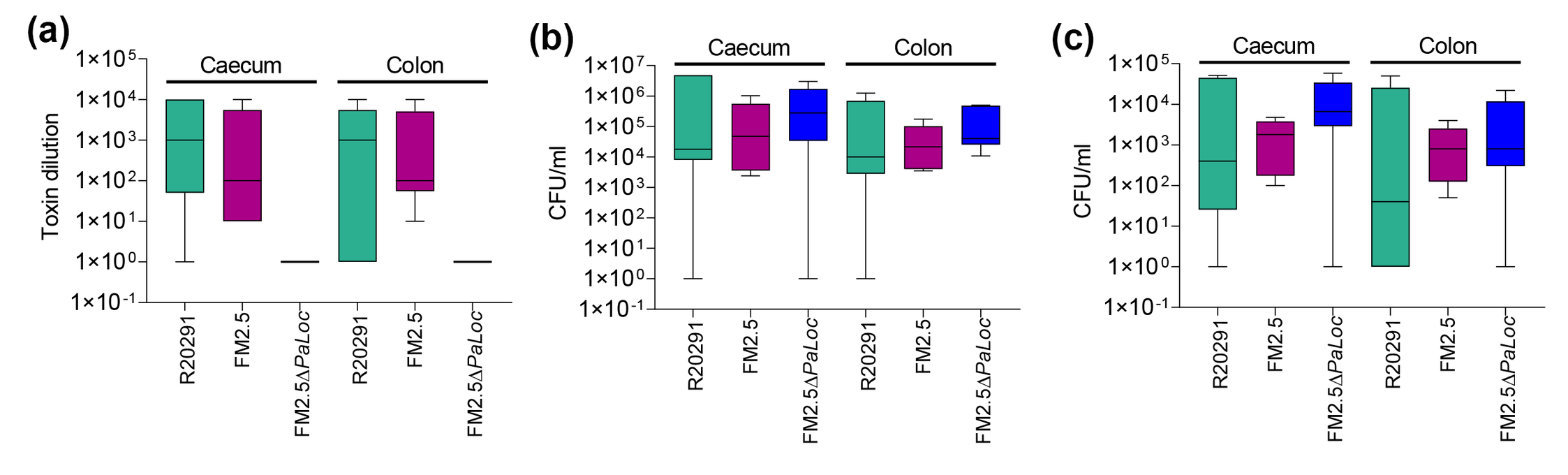

Supplement: S5 Fig — Female C57/Bl6 mice were challenged with spores of R20291 (green), FM2.5 (purple) or FM2.5ΔPaLoc (blue). (a) Toxin activity of caecal and colonic content was determined as the reciprocal of last dilution in which cytoplasmic changes to challenged were observed. Statistical significance is indicated: ns—not significant. (b) At the experimental endpoint (96 hpi), total C. difficile counts were enumerated by colony counts from the caecum and colon contents from individual animals (n = 5 per group). (c) Spores were obtained from caecum and colon material and enumerated by colony counts. Vegetative cells were killed by heating samples at 65 °C for 20 min prior to plating to ensure only spores remained. (TIF) [file ppat.1011015.s005.tif]

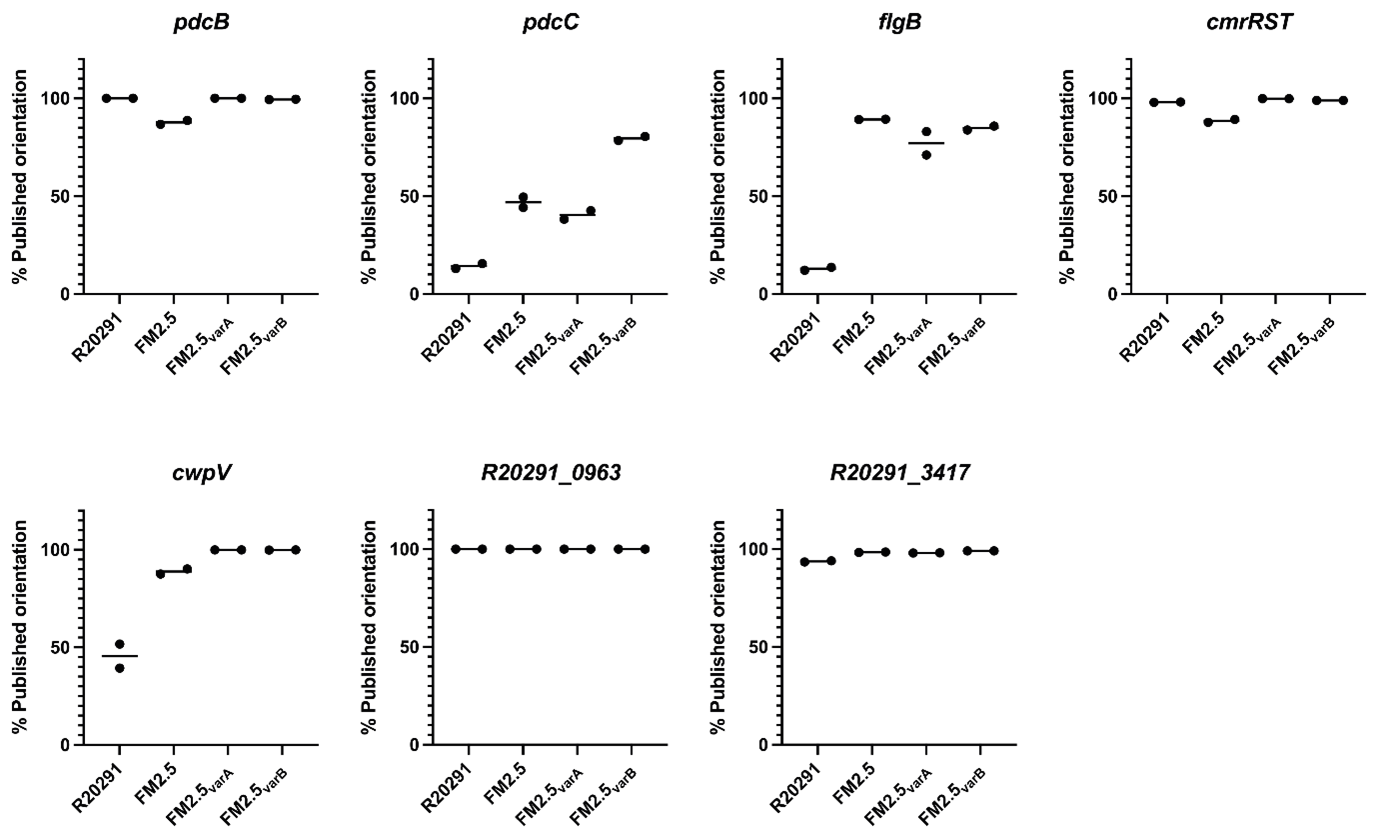

Supplement: S6 Fig — To determine if differences in pathogenesis could be linked to a dominance of switch orientations of the 7 known phase switches in C. difficile, their orientation in all four strains was evaluated using qPCR, using the method described in [44]. This shows that the differences in virulence observed between FM2.5varA and FM2.5varB does not appear to link to orientation of these switches. Mixed populations representing 100–200 independent colonies were pooled, genomic DNA was extracted and the orientations of the seven switches was determined using a previously described orientation-specific qPCR assay. The gene immediately downstream of the switch and presumed or confirmed to be controlled by the switch, is indicated above each graph. The percentages of each switch found to be in the same orientation as in the published R20291 reference genome are shown. Measurements were performed in duplicate with the mean indicated by the horizontal line. Although variation in orientation between strains is observed for the switches upstream of pdcC, flgB and cwpV these differences do not correlate with observed differences in virulence. (TIF) [file ppat.1011015.s006.tif]
