# Supplementary material for: An intact S-layer is advantageous to Clostridioides difficile within the host
Source: PLoS Pathog. 2023 Jun 29;19(6):e1011015. doi: 10.1371/journal.ppat.1011015 (PMC10310040; doi:10.1371/journal.ppat.1011015)
Supplement: S1 Table — Data collection and refinement statistics for structure determination of SlpAvarB. Data collected at beamline I24, Diamond Light Source. Values in parenthesis correspond to the highest resolution shell. R-work = Σ ||Fobs|—|Fcalc|| / Σ |Fobs| R-free = Σ ||Fobs|—|Fcalc|| / Σ |Fobs| for 5% reflections excluded from refinement I/sigmaI = I / σ(I) CC1/2 = Σ (Fobs1 * Fobs2*) / (√(Σ |Fobs1|^2) * √(Σ |Fobs2|^2)). (DOCX) [file ppat.1011015.s007.docx]

**Table S1.** **Data collection and refinement statistics**

|  | H/L SlpA_varB_ |
| --- | --- |
| **Data collection** |  |
| Space group | *C*2 |
| Cell dimensions |  |
| *a*, *b*, *c* (Å) | 108.9 131.3 138.3 |
| α, β, *γ* (°) | 90.0, 108.8, 90.0 |
| Wavelength (Å) | 0.7085 |
| Resolution (Å) | 36.23 – 2.90  (3.00 – 2.90) |
| I/σI | 8.0 (2.2) |
| CC1/2 | 0.978 (0.526) |
| Completeness (%) | 98 (87) |
| Redundancy | 2.0 (2.0) |
|  |  |
| **Refinement** |  |
| Resolution (Å) | 36.23 – 2.90 |
| No. reflections | 40000 |
| *R*_work_ / *R*_free_ | 24.6/25.2 |
| No. atoms |  |
| Protein | 8352 |
| Ligand/ion | 2 |
| Water | 32 |
| *B*-factors (Å^2^) |  |
| Protein | 54.4 |
| Ligand/ion | 39.9 |
| Water | 36.9 |
| Ramachandran % |  |
| favoured | 96.8 |
| allowed | 3.0 |
| outliers | 0.2 |
| R.m.s. deviations |  |
| Bond lengths (Å) | 0.002 |
| Bond angles (°) | 0.52 |
| PDB ID | 8BBY |

*Values in parentheses are for highest-resolution shell.
