# Supplementary material for: An intact S-layer is advantageous to Clostridioides difficile within the host
Source: PLoS Pathog. 2023 Jun 29;19(6):e1011015. doi: 10.1371/journal.ppat.1011015 (PMC10310040; doi:10.1371/journal.ppat.1011015)
Supplement: S2 Table — List of interactions between neighbouring molecules within the S-layer tiling for SlpAvarB and previously determined SlpACD630 and SlpARΔD2. Interacting atoms and the corresponding residue, together with the bond distance and interaction type (H—hydrogen bond; S—salt bridge) are listed, as determined in PISA and verified in each model using Coot (see material and methods for details). Numbers in parenthesis refer to the number of the neighbouring molecule, as defined in Fig 3b. L—SLPL; H—SLPH. Interactions seen in SlpAvarB not previously described in other models are highlighted in crimson. (DOCX) [file ppat.1011015.s008.docx]

**Table S2.** **Interactions across S-layer tiling**

| **SlpA_CD630_ (PDB ID 7ACY)** | | | | | | **SlpA_RΔD2_ (PDB ID 7ACZ)** | | | | | | **SlpA_varB_ (PDB ID 8BBY)** | | | | | | | | |  |
| --- | --- | --- | --- | --- | --- | --- | --- | --- | --- | --- | --- | --- | --- | --- | --- | --- | --- | --- | --- | --- | --- |
| **Residue** | **Atom** | **Residue** | **Atom** | **Type** | **Distance (Å)** | **Residue** | **Atom** | **Residue** | **Atom** | **Type** | **Distance (Å)** | | **Residue** | | **Atom** | **Residue** | | **Atom** | **Type** | **Distance (Å)** | |
| **D1-D1** | | | | | | | | | | | | | | | | | | | | | |
| F38_L(1)_ | O | N66_L(4)_ | N^δ2^ | H | 3.0 | A49_L(1)_ | O | Q70_L(4)_ | N^ε2^ | H | 3.3 | |  | |  |  | |  |  |  | |
| G41_L(1)_ | O | N66_L(4)_ | N^δ2^ | H | 3.3 | S50_L(1)_ | O^γ^ | S50_L(4)_ | O^γ^ | H | 2.5 | |  | |  |  | |  |  |  | |
| N66_L(1)_ | N^δ2^ | F38_L(4)_ | O | H | 2.9 | Q70_L(1)_ | N^ε2^ | A49_L(4)_ | O | H | 3.1 | | No D1-D1 interactions detected | | | | | | | | |
| N66_L(1)_ | N^δ2^ | G41_L(4)_ | O | H | 3.3 |  |  |  |  |  |  | |  | |  |  | |  |  |  | |
| T70_L(1)_ | O^γ1^ | Q71_L(4)_ | O^ε1^ | H | 3.1 |  |  |  |  |  |  | |  | |  |  | |  |  |  | |
| Q71_L(1)_ | O^ε1^ | T70_L(4)_ | O^γ1^ | H | 3.0 |  |  |  |  |  |  | |  | |  |  | |  |  |  | |
| **D1-D2** | | | | | | | | | | | | | | | | | | | | | |
| N208_L(1)_ | N^δ2^ | E295_L(6)_ | O^ε1^ | H | 2.8 | No D2 present | | | | | | | D2 not traceable | | | | | | | | |
| **D2-D1** | | | | | | | | | | | | | | | | | | | | | |
| E295_L(1)_ | O^ε1^ | N208_L(6)_ | N^δ2^ | H | 3.0 | No D2 present | | | | | | | D2 not traceable | | | | | | | | |
| K232_L(1)_ | N^ζ^ | E295_L(6)_ | O^ε2^ | S | 3.7 |  |  |  |  |  |  | |  |  | |  |  | |  |  | |
| **D1-CWB2_1_** | | | | | | | | | | | | | | | | | | | | | |
| D77_L(1)_ | O^δ1^ | K159_H(2)_ | N^ζ^ | S | 3.5 | G81_L(1)_ | N | T133_H(2)_ | O^γ1^ | H | 3.9 | | D84_L(1)_ | | O^δ1^ | K136_H(2)_ | | N^ζ^ | S | 3.1 | |
| D77_L(1)_ | O^δ2^ | K159_H(2)_ | N^ζ^ | S | 3.3 |  |  |  |  |  |  | | D84_L(1)_ | | O^δ1^ | K160_H(2)_ | | N^ζ^ | S | 3.2 | |
| G78_L(1)_ | N | T132_H(2)_ | O^γ1^ | H | 3.8 |  |  |  |  |  |  | |  | |  |  | |  |  |  | |
| **D1-CWB2_2_** | | | | | | | | | | | | | | | | | | | | | |
| N13_L(1)_ | N^δ2^ | S231_H(5)_ | O | H | 2.9 | S19_L(1)_ | O^γ^ | D241_H(5)_ | O^δ2^ | H | 3.7 | | K20_L(1)_ | | N^ζ^ | D267_H(5)_ | | O^δ2^ | H | 3.5 | |
| N13_L(1)_ | N^δ2^ | S231_H(5)_ | O^γ^ | H | 2.6 |  |  |  |  |  |  | | K22_L(1)_ | | N^ζ^ | D244_H(5)_ | | O^δ2^ | H | 3.8 | |
| K17_L(1)_ | N^ζ^ | D184_H(5)_ | O^δ1^ | S | 3.0 |  |  |  |  |  |  | | K22_L(1)_ | | N^ζ^ | D245_H(5)_ | | O^δ1^ | S | 3.0 | |
| K20_L(1)_ | N^ζ^ | D182_H(5)_ | O | H | 3.2 |  |  |  |  |  |  | | K23_L(1)_ | | N^ζ^ | D266_H(5)_ | | O^δ1^ | S | 3.7 | |
|  |  |  |  |  |  |  |  |  |  |  |  | | K23_L(1)_ | | N^ζ^ | D267_H(5)_ | | O^δ1^ | S | 3.2 | |
|  |  |  |  |  |  |  |  |  |  |  |  | | H71_L(1)_ | | N^δ1^ | D244_H(5)_ | | O^δ2^ | S | 3.6 | |
|  |  |  |  |  |  |  |  |  |  |  |  | | H71_L(1)_ | | N^ε2^ | D244_H(5)_ | | O^δ2^ | S | 3.7 | |
| **LID-CWB2_1_** | | | | | | | | | | | | | | | | | | | | | |
| K315_L(1)_ | N | I130_H(2)_ | O | H | 3.3 | E167_L(1)_ | O | N132_H(2)_ | N^δ1^ | H | 3.1 | |  | |  |  | |  |  |  | |
| E318_L(1)_ | O^ε2^ | K119_H(2)_ | N^ζ^ | H | 3.3 | K169_L(1)_ | N | I131_H(2)_ | O | H | 2.9 | |  | |  |  | |  |  |  | |
| E318_L(1)_ | O^ε2^ | K119_H(2)_ | N^ζ^ | S | 3.3 | K169_L(1)_ | O | I131_H(2)_ | N | H | 2.6 | |  | |  |  | |  |  |  | |
|  |  |  |  |  |  | K169_L(1)_ | N^ζ^ | D156_H(2)_ | O | H | 2.8 | |  | |  |  | |  |  |  | |
|  |  |  |  |  |  | T172_L(1)_ | O | K120_H(2)_ | N^ζ^ | S | 3.8 | |  | |  |  | |  |  |  | |
| **LID-CWB2_2_** | | | | | | | | | | | | | | | | | | | | | |
| S260_L(1)_ | N | E253_H(5)_ | O^ε2^ | H | 3.3 | K107_L(1)_ | N^ζ^ | D237_H(5)_ | O^δ2^ | S | 2.5 | |  | |  |  | |  |  |  | |
| S260_L(1)_ | O^γ^ | E253_H(5)_ | O^ε2^ | H | 3.1 | K107_L(1)_ | N^ζ^ | D237_H(5)_ | O^δ1^ | S | 3.6 | |  | |  |  | |  |  |  | |
| S256_L(1)_ | O^δ2^ | E257_H(5)_ | O^γ^ | H | 3.8 | S384_H(1)_ | O^γ^ | S384_H(3)_ | O^γ^ | H | 3.9 | |  | |  |  | |  |  |  | |
|  |  |  |  |  |  | K385_H(1)_ | N^ζ^ | S384_H(3)_ | O | H | 3.8 | |  | |  |  | |  |  |  | |
| **HID-CWB2_1_** | | | | | | | | | | | | | | | | | | | | | |
| D9_H(1)_ | O^δ1^ | T132_H(2)_ | O^γ1^ | H | 3.2 | D8_H(1)_ | O^δ2^ | N132_H(2)_ | N^δ2^ | H | 3.6 | | N10_H(1)_ | | N^δ2^ | T133_H(2)_ | | O^γ1^ | H | 3.3 | |
| D9_H(1)_ | O | T132_H(2)_ | N | H | 3.6 | N10_H(1)_ | N^δ2^ | T133_H(2)_ | O^γ1^ | H | 2.9 | |  | |  |  | |  |  |  | |
| K31_L(1)_ | O | I130_H(2)_ | N | H | 3.0 | N10_H(1)_ | O | T133_H(2)_ | O^γ1^ | H | 3.7 | |  | |  |  | |  |  |  | |
| R31_L(1)_ | N^ε1^ | S126_H(2)_ | O | H | 3.7 |  |  |  |  |  |  | |  | |  |  | |  |  |  | |
| E31_L(1)_ | O^ε1^ | G129_H(2)_ | N | H | 3.4 |  |  |  |  |  |  | |  | |  |  | |  |  |  | |
| **HID-CWB2_2_** | | | | | | | | | | | | | | | | | | | | | |
| K20_H(1)_ | N^ζ^ | D256_H(3)_ | O^δ1^ | S | 2.6 | K21_H(1)_ | N^ζ^ | D266_H(3)_ | O^δ1^ | S | 3.0 | | D27_H(1)_ | | O^δ2^ | K287_H(3)_ | | N^ζ^ | S | 3.9 | |
| K20_H(1)_ | N^ζ^ | D256_H(3)_ | O^δ2^ | S | 3.4 | K21_H(1)_ | N^ζ^ | D266_H(3)_ | O^δ2^ | S | 3.4 | |  | |  |  | |  |  |  | |
| K20_H(1)_ | N^ζ^ | T262_H(3)_ | O^γ1^ | H | 3.4 | K23_H(1)_ | N^ζ^ | D274_H(3)_ | O^δ1^ | S | 3.5 | |  | |  |  | |  |  |  | |
| K22_H(1)_ | N^ζ^ | T262_H(3)_ | O | H | 2.8 | N10_H(1)_ | O | T133_H(2)_ | N | H | 3.82 | |  | |  |  | |  |  |  | |
| K22_H(1)_ | N^ζ^ | D264_H(3)_ | O^δ1^ | S | 3.0 | K23_H(1)_ | N^ζ^ | S272_H(3)_ | O^γ^ | H | 2.9 | |  | |  |  | |  |  |  | |
| K22_H(1)_ | N^ζ^ | D264_H(3)_ | O^δ2^ | S | 2.9 | D24_H(1)_ | O^δ1^ | S272_H(3)_ | O^γ^ | H | 2.8 | |  | |  |  | |  |  |  | |
| **HID-CWB2_3_** | | | | | | | | | | | | | | | | | | | | | |
|  |  |  |  |  |  |  |  |  |  |  |  | | **Y35_H(1)_** | | **OH** | **S377_H(3)_** | | **O^γ^** | **H** | **3.0** | |
| **CWB2_1_-HID** | | | | | | | | | | | | | | | | | | | | | |
| S126_H(1)_ | O^γ^ | Y38_H(2)_ | O | H | 3.4 | T133_H(1)_ | O^γ1^ | N10_H(2)_ | O | H | 3.9 | | T133_H(1)_ | | O^γ1^ | E9_H(2)_ | | O^ε2^ | H | 3.8 | |
| N131_H(1)_ | N^δ2^ | D9_H(2)_ | O^δ2^ | H | 3.5 | T133_H(1)_ | O^γ1^ | N10_H(2)_ | O^δ1^ | H | 2.9 | |  | |  |  | |  |  |  | |
| T132_H(1)_ | N | D9_H(2)_ | O | H | 3.3 |  |  |  |  |  |  | |  | |  |  | |  |  |  | |
| T132_H(1)_ | O^γ1^ | D9_H(2)_ | O | H | 3.8 |  |  |  |  |  |  | |  | |  |  | |  |  |  | |
| **CWB2_2_-HID** | | | | | | | | | | | | | | | | | | | | | |
| T262_H(1)_ | O^γ1^ | K20_H(3)_ | N^ζ^ | H | 3.4 | D266_H(1)_ | O^δ1^ | K21_H(3)_ | N^ζ^ | S | 3.6 | | D274_H(1)_ | | O^δ1^ | K23_H(3)_ | | N^ζ^ | S | 3.5 | |
| T262_H(1)_ | O | K22_H(3)_ | N^ζ^ | H | 2.6 |  |  |  |  |  |  | | R275_H(1)_ | | O | K23_H(3)_ | | N^ζ^ | H | 3.6 | |
| D256_H(1)_ | O^δ1^ | K20_H(3)_ | N^ζ^ | S | 2.5 |  |  |  |  |  |  | |  | |  |  | |  |  |  | |
| D256_H(1)_ | O^δ2^ | K20_H(3)_ | N^ζ^ | S | 3.3 |  |  |  |  |  |  | |  | |  |  | |  |  |  | |
| D264_H(1)_ | O^δ1^ | K22_H(3)_ | N^ζ^ | S | 3.1 |  |  |  |  |  |  | |  | |  |  | |  |  |  | |
| D264_H(1)_ | O^δ2^ | K22_H(3)_ | N^ζ^ | S | 3.8 |  |  |  |  |  |  | |  | |  |  | |  |  |  | |
| **CWB2_1_-CWB2_3_** | | | | | | | | | | | | | | | | | | | | | |
| N123_H(1)_ | N^δ2^ | D64_H(2)_ | O^δ2^ | H | 3.0 |  |  |  |  |  |  | | K126_H(1)_ | | N^ζ^ | D65_H(2)_ | | O^δ1^ | S | 3.2 | |
| K125_H(1)_ | N^ζ^ | D64_H(2)_ | O^δ1^ | S | 3.7 |  |  |  |  |  |  | | K126_H(1)_ | | N^ζ^ | R72_H(2)_ | | O | H | 2.9 | |
| K125_H(1)_ | N^ζ^ | D64_H(2)_ | O^δ2^ | S | 3.1 |  |  |  |  |  |  | |  | |  |  | |  |  |  | |
| **CWB2_2_-CWB2_3_** | | | | | | | | | | | | | | | | | | | | | |
| D282_H(1)_ | O^δ2^ | K347_H(3)_ | N^ζ^ | S | 2.8 | K126_H(1)_ | N^ζ^ | D65_H(2)_ | O^δ1^ | S | 3.3 | | N124_H(1)_ | | N^δ2^ | D65_H(2)_ | | O^δ2^ | H | 2.9 | |
| D287_H(1)_ | O^δ2^ | K347_H(3)_ | N^ζ^ | S | 3.8 |  |  |  |  |  |  | |  | |  |  | |  |  |  | |
| **CWB2_3_-CWB2_1_** | | | | | | | | | | | | | | | | | | | | | |
| D64_H(1)_ | O^δ2^ | N123_H(2)_ | N^δ1^ | H | 3.2 | D266_H(1)_ | O^δ1^ | K21_H(3)_ | N^ζ^ | H | 3.6 | | D65_H(1)_ | | O^δ1^ | K126_H(2)_ | | N^ζ^ | S | 3.3 | |
| D64_H(1)_ | O | K125_H(2)_ | N^ζ^ | H | 3.6 |  |  |  |  |  |  | | R72_H(1)_ | | O | K126_H(2)_ | | N^ζ^ | H | 2.7 | |
| D64_H(1)_ | O^δ1^ | K125_H(2)_ | N^ζ^ | S | 3.6 |  |  |  |  |  |  | |  | |  |  | |  |  |  | |
| D64_H(1)_ | O^δ2^ | K125_H(2)_ | N^ζ^ | S | 3.0 |  |  |  |  |  |  | |  | |  |  | |  |  |  | |
| **CWB2_3_-CWB2_2_** | | | | | | | | | | | | | | | | | | | | | |
| K347_H(1)_ | N^ζ^ | D282_H(3)_ | O^δ1^ | S | 3.1 |  |  |  |  |  |  | |  | |  |  | |  |  |  | |
| K347_H(1)_ | N^ζ^ | D282_H(3)_ | O^δ2^ | S | 2.9 |  |  |  |  |  |  | |  | |  |  | |  |  |  | |
| K347_H(1)_ | N^ζ^ | D287_H(3)_ | O^δ1^ | S | 2.7 |  |  |  |  |  |  | |  | |  |  | |  |  |  | |
| K347_H(1)_ | N^ζ^ | D287_H(3)_ | O^δ2^ | S | 2.9 |  |  |  |  |  |  | |  | |  |  | |  |  |  | |
| **CWB2_3_-CWB2_3_** | | | | | | | | | | | | | | | | | | | | | |
| S335_H(1)_ | O^γ^ | N338_H(3)_ | N^δ2^ | H | 3.1 |  |  |  |  |  |  | |  | |  |  | |  |  |  | |
| S335_H(1)_ | O^γ^ | K367_H(3)_ | N^ζ^ | H | 2.6 |  |  |  |  |  |  | |  | |  |  | |  |  |  | |
| N338_H(1)_ | N^δ2^ | S335_H(3)_ | O^γ^ | H | 3.0 |  |  |  |  |  |  | |  | |  |  | |  |  |  | |
| S342_H(1)_ | O^γ^ | V339_H(3)_ | O | H | 3.6 |  |  |  |  |  |  | |  | |  |  | |  |  |  | |
| K343_H(1)_ | NZ | L371_H(3)_ | O | H | 3.4 |  |  |  |  |  |  | |  | |  |  | |  |  |  | |
| L371_H(1)_ | O | K343_H(3)_ | N^ζ^ | H | 3.5 |  |  |  |  |  |  | |  | |  |  | |  |  |  | |

Numbers in parenthesis refer to the number of the neighbouring molecule, as defined in Fig. 3b. L - SLPL; H – SLPH. Interactions seen in SlpA_varB_ not previously described are highlighted in crimson
