# Supplementary material for: An intact S-layer is advantageous to Clostridioides difficile within the host
Source: PLoS Pathog. 2023 Jun 29;19(6):e1011015. doi: 10.1371/journal.ppat.1011015 (PMC10310040; doi:10.1371/journal.ppat.1011015)
Supplement: S3 Table — Oligonucleotide primers used for PCR and qPCR. Sequences in lowercase indicate homology regions for Gibson assembly. All oligonucleotides were purchased from Eurofins Genomics Europe. (DOCX) [file ppat.1011015.s009.docx]

**Table S3. Oligonucleotides used in this study**

| **Primer name** | **Sequence** | **Purpose** |
| --- | --- | --- |
| RF110 | GACATAACTGCAGCACTACTTG | Amplification of 478 bp of *slpA* centred on the FM2.5 mutation. |
| RF111 | CAGGATTAACAGTATTAGCTTCTGC | Amplification of 478 bp of *slpA* centred on the FM2.5 mutation. |
| RF311 | TAGGGTAACAAAAAACACCG | Linearisation of pJAK112. |
| RF312 | CCTTTTTGATAATCTCATGACC | Linearisation of pJAK112. |
| RF920 | cgtagaaatacggtgttttttgttaccctaTGGAATTTAGATATAAAAACCAATTC | Amplification of *PaLoc* homology arms. Homology regions for Gibson Assembly are shown in lowercase. |
| RF921 | atttattttggtgtgGACAACATTGGAATTAAATCAG | Amplification of *PaLoc* homology arms. Homology regions for Gibson Assembly are shown in lowercase. |
| RF922 | aattccaatgttgtcCACACCAAAATAAATGCC | Amplification of *PaLoc* homology arms. Homology regions for Gibson Assembly are shown in lowercase. |
| RF923 | gggattttggtcatgagattatcaaaaaggCCCAACTATGGAAAAACC | Amplification of *PaLoc* homology arms. Homology regions for Gibson Assembly are shown in lowercase. |
| RF2193 | ACACTCTTTCCCTACACGACGCTCTTCCGATCTCTACTTGTAGCTACTTTTATTGCAC | Amplification of 330 bp of *slpA* for amplicon sequencing. |
| RF2194 | GACTGGAGTTCAGACGTGTGCTCTTCCGATCTCAAGGATATACAGTAGTACAGAGC | Amplification of 330 bp of *slpA* for amplicon sequencing. |

| Primers for orientation-specific qPCR: | | |
| --- | --- | --- |
| RF2432 | CGCAATTATTTGTTTTTCATATGGATAAAATTGG | *cwpV* - PUB |
| RF2433 | GATTTTTATGTTAATGAATTGTTATAAAAAACATGG | *cwpV* - PUB |
| RF2434 | GGTAAGTTTGATTTTTATGTTAATGAATTG | *cwpV* - INV |
| RF2435 | CAGTTTGTGCACTAGCTATGCCTGC | *cwpV* - INV |
| RF2436 | CATTTCTAAGAAATATCCTAACATAAAAACAAAA | *pdcB* - PUB |
| RF2437 | CGATTACACTACAGAATTAGAATGTCAATG | *pdcB* - PUB |
| RF2438 | GTTAAAAATTTAAGATATCTTTTCAGTATAATGGA | *pdcB* - INV |
| RF2439 | CATTTCTAAGAAATATCCTAACATAAAAACAAAA | *pdcB* - INV |
| RF2440 | GATTTGTCGAAACCATTGTAATAAGA | *pdcC* - INV |
| RF2441 | CAATAGTTAAGACAATGAATATGCTACATTCT | *pdcC* - INV |
| RF2442 | CAATAGTTAAGACAATGAATATGCTACATTCT | *pdcC* - PUB |
| RF2443 | GTAAATTCCTCATAAAAATTTCCTCCCA | *pdcC* - PUB |
| RF2444 | GTTTTCTTACCAAAGTGATACATTATTATATTAATG | *flgB* - PUB |
| RF2445 | GCTATTGTCTGACTTCTTAAATTAGTTGCAT | *flgB* - PUB |
| RF2446 | CATTAATATAATAATGTATCACTTTGGTAAGAAAAC | *flgB* - INV |
| RF2447 | GCTATTGTCTGACTTCTTAAATTAGTTGCAT | *flgB* - INV |
| RF2448 | GTAAATTAAGATGTATTTCATTTCTCAAAAATATCCT | CDR20291_0963 - PUB |
| RF2449 | GCTTTTATCGCAAGTTTGTTTTAAATGAC | CDR20291_0963 - PUB |
| RF2450 | GTAAATTAAGATGTATTTCATTTCTCAAAAATATCCT | CDR20291_0963 - INV |
| RF2451 | GTAAAGTTTATAAAATCTGAAAAGCTCAAGA | CDR20291_0963 - INV |
| RF2452 | CTAGCCAATAGACAAGTTTCTAGAAAAATA | *cmrRST* - PUB |
| RF2453 | GAACAATTCTTGAATATTGTATTGAACATTAAGA | *cmrRST* - PUB |
| RF2454 | GGAGATATATGGAGTTAGTGGTGCAA | *cmrRST* - INV |
| RF2455 | CTAGCCAATAGACAAGTTTCTAGAAAAATA | *cmrRST* - INV |
| RF2456 | GTACAGAAGTTACCCAGAAGCTTGT | CDR20291_3417 - PUB |
| RF2457 | TCCCCGCAATGGATGTTTTTTAATTCATC | CDR20291_3417 - PUB |
| RF2458 | GTACAGAAGTTACCCAGAAGCTTGT | CDR20291_3417 - INV |
| RF2459 | TCCCAATTTAAATGTAGAGGTCATCAAT | CDR20291_3417 - INV |
| RF2460 | TCATTACCAGGTGTAGCAGTGAATGC | *rpoA* |
| RF2461 | TGATAGAGCATGGTCCTTGAGCTTCT | *rpoA* |
