# Supplementary material for: An intact S-layer is advantageous to Clostridioides difficile within the host
Source: PLoS Pathog. 2023 Jun 29;19(6):e1011015. doi: 10.1371/journal.ppat.1011015 (PMC10310040; doi:10.1371/journal.ppat.1011015)
Supplement: Attachment — Submitted filename: Final response to reviewers.docx [file ppat.1011015.s011.docx]

Response to Reviewers comments

We would like to thank the reviewers for their time and effort reviewing our manuscript. We believe the changes, as outlined below, will address their concerns, and thank them for their suggestions, which have helped to further clarify the message of the paper.

Reviewer #1: 1) The authors state levels of significance in their figure legends, but the figures themselves largely ignore significance. It is hard to tell if the values presented are significantly different, or if the authors simply forgot to label the figures properly.

Firstly, we wish to apologise to the reviewers, as an earlier version of the figure, lacking indicators of significance, was uploaded in error. In addition, considering the reviewers’ comments, Fig. 1 has now undergone a major transformation, which we believe provides a clearer narrative by focussing on data from the caecal tissue (an equivalent analysis of colonic material is now provided in Fig. S1). Additional data has also been included which should address highlighted concerns with respect to statistical power of the work presented.

In the earlier version of the paper, we chose to present data for caecal and colonic material from a single representative experiment, in which groups of 5 mice per strain were used. However, these experiments have been performed multiple times and data from other experiments have now been included to enhance statistical power. The number of samples used at each time point is indicated within the figure legend. Please note that variation in numbers of samples for some time points, particularly 48 and 72 hpi, reflects the fact that collection of material was difficult, or samples are unavailable; faecal samples at 48 hpi, when animals infected with R20291 have diarrhoea and tissue at 72 hpi when animals had to be culled prior to this time point because of severe disease. An explanation for differences in numbers, used in the figures and analysis, is provided in the text and figure legend descriptions.

As an example, the FM2.5 strain has a ~1 log10 reduction in CFU in the mouse feces 24 - 72 hpi and this defect is much larger in the cecum and the colon. But if these differences are not significant (and the authors stated on line 153 that it is a trend for less recovery), then this reduces the impact of the findings.

Inclusion of the additional data has allowed us to show that while there was no statistical difference in total number (spores and vegetative cells) of *C. difficile* recovered from faeces of animals infected with R20291 or FM2.5 at 24 hpi, the number of spores recovered from faeces differed significantly. These data agree with the previous published observation that FM2.5 sporulates less efficiently.

2) Is the reason that fewer spores are recovered in Fig. 4D is that what spores are formed are sensitive to heat? The authors enumerate using 56C as their selection for spores vs. vegetative cells. Given the differences in transcripts related to sporulation, it is possible that they either 1: do not make spores or 2: what spores are made cannot be enumerated.

We would like to apologise for the lack of clarity explaining the methodology used in this experiment, which could lead to misunderstanding of the data. By including assessment of the nucleic acid recovered from the spores used to challenge the animals, we hoped to highlight the fact that low numbers of variants were present in the original inoculum.

However, during *in vivo* analysis, we didn’t enumerate or purify spores, rather, DNA was extracted directly from faecal pellets for subsequent amplicon amplification and sequencing, making quantification of sequence variants unbiased toward recovery of spores or vegetative cells. This figure highlights that the variants in the inoculum appear to have a significant selective advantage within the animal. The description of the unbiased analysis is provided in the methods, but we have added an additional sentence for clarification. For clarity, this Fig. 4D, has been moved to support the data shown in Fig. 2. This also avoids any unnecessary confusion with the other data presented in Fig. 4, which refers to the experiments using strains lacking the PaLoc. Panel 4C in the original Fig. 4, which was included to indicate times of faecal recovery for this analysis, has been removed.

3) Figure 5. The comparison for statistical significance should not be to the toxin control. Because strains are being compared, it is more appropriate to compare within the strains - e.g., compare to R20291, etc.

Thank you for this observation. The analysis has now been changed to reflect this comment, which importantly still shows that FM2.5 appears to make significantly less toxin than R20291 at 36 h but not by 72 h.

Reviewer #2: The naming of the reversion mutants RvA and RvB is generic and not indicative of the gene reversion being referenced. The authors should instead adhere to standard prokaryotic mutation nomenclature (see ASM).

We thank the reviewer for this suggestion and provide the standard prokaryotic nomenclature at the first point of their use in the text, together with a shortened name offered for use in the text and on figures. We hope this shortened name for the strains acknowledges concerns from the reviewers regarding the term ‘revertant’. Instead, we have chosen to use the term variant, abbreviated to **var**, for strains showing variation in the annotated sequence of *slp*A. As they derive from the previously described strain FM2.5, they are now referred to FM2.5_varA_, FM2.5_varB_, etc. and the SlpA proteins produced annotated as SlpA_varA_, SlpA_varB_ and so on.

The animal experiments and subsequent experimental analyses throughout the manuscript are underpowered to achieve statistically significant results. Some experiments were performed with as few as three animals and a single replicate (e.g., toxin activity, CFU). Other experiments include 2-3 fold more animals infected with the FM2.5 strain than other strains, which does not appear to be scientifically justified.

As described above, the data for Fig. 1 (and Fig. S1 supplement) have been revised to include data from additional experiments. The number of samples included in each analysis is provided in the figure legends. The number of samples analysed does vary between strains and samples but reflects difficulties in recovery of some of the samples. For example, faeces were difficult to recover from animals infected with R20291 48 h post infection as many had diarrhoea at this time point. In contrast, FM2.5 infected animals showed less disease, allowing more samples to be recovered. Similarly, differences at 72 h reflect the fact that several animals infected with R20291 had to be culled before this time point because of disease severity. A description of the reason for these differences in sample numbers has now been included in the manuscript.

The authors delete the PaLoc (toxin locus) from the FM2.5 slpA mutant and compare it in mouse infections relative to the wildtype and FM2.5 (Fig 4). The requirement of the toxins for virulence is well established, so it is not obvious what question is being asked or answered with this experiment.

The aim of this experiment was to establish whether the selection of S-layer variants was dependent or independent of toxin production, which as the reviewer points out is a known requirement for virulence. Given that we have previously shown FM2.5 to produce less toxin that would result in less tissue damage and inflammation, we wanted to determine whether, in the absence of toxin, selection pressure for recovery of the intact S-layer would be reduced. Fig. 4 has now been modified to provide a clearer focus on this question. The text has also been modified to clarify this point.

Further, panel C shows the weight change for the FM2.5 without control comparisons, which lacks the context required for evaluation.

Panel C was originally included to indicate the times of faecal collection required for the unbiased sequencing of the variant *slpA* allele. As the times are indicated in Fig. 2G, this figure has now been removed to provide a clearer narrative.

RNA-seq analysis (Fig 6) uses the FM2.5 mutant as the control comparator, rather than the wild-type strain. This comparison is not conventional and it is difficult to interpret.

The choice of FM2.5 as a comparator was made on the basis that the variants were more closely related to FM2.5 than R20291. However, the data has been reanalysed and is presented in the more conventional manner, as suggested by the reviewer. We thank the reviewer for this observation as we believe it helps with the interpretation and clarity of the data presented.

Fig 2 images are not to scale, which makes it difficult to compare the sizes of the colonies and the relative difference between the mutant and control.

These figures have been regenerated and are now equivalent in size, allowing the relative differences in colony size to be more easily observed and compared between the different plates.

The abstract is long and diffuse with tangential mentions of discussion points. A more focused account of the primary findings would be helpful to the readers.

We appreciate the reviewer’s comments and have rewritten the abstract to provide clarity and focus.

Reviewer #3: 1. My biggest concern is the potential for different the known phase variable switches in C. difficile to be in different phases in the revertants. Could this explain the colony size difference in some of the revertants? Have the authors considered reconstructing a strain with the revertant mutants to show they are responsible for the changes in virulence and gene expression? Alternatively complementing the FM2.5 mutant with RvA vs RvC to see if they recapitulate the most interesting phenotypes.

With respect to colony size, we believe the differences observed are not reflective of phase switching as the colony size appears stable on subsequent passage, which would be unlikely if this phenotype reflected such changes. A sentence has been included in the text to reflect this observation.

Phase variation is a complex issue that isn’t easily resolvable, as the very heterologous nature of these changes means that the switches are likely to be in different orientations even within clones of the parental strain, R20291. That said, we cannot dismiss this possibility, as the differences in pathogenesis could be linked to a dominance of switch orientations, affecting several associated traits. To address this issue, we have assessed the orientation of the 7 known switches in all four strains using qPCR on genomic DNA from a mixed population of around 1-200 colonies of each strain. The method used followed faithfully that described in Reyes Ruiz et al PLoS Pathog. 2022 Jul 5;18(7):e1010677. These data provide a snapshot of the orientation of the switches at time of testing, showing that for at least 4 of the switches, there is no difference in orientation within R20291 and the variant strains. In the remaining 3 switches, for at least 2 of these, the data generated contradicts the phenotypes we have observed experimentally. For example, for the *cwpV* switch, data reported (Emerson et al Mol Microbiol. 2009 Nov;74(3):541-56) suggests the phase switch is only on in around 5% of the R20291 population (nearly 50% here). In contrast, *cwpV* has been reported to be highly expressed in FM2.5, an observation additionally supported by RNA-seq data generated and reported in this paper.

In addition, changes in *flgB* orientation help support evidence that phase variation is not responsible for the observed changes in virulence with the variants, as both show the same orientation as FM2.5, yet for FM2.5_varA_ this was not associated with an attenuated phenotype or any downregulation in expression of toxin as determined by RNA-seq. Another point to note, is that the switch orientation for wild type R20291 observed here is the reverse of that reported by Reyes Ruiz *et. al.*. We believe this may reflect that the studies may have been performed on different lineages of the parental R20291, which have been previously described (Monteford et al, Anaerobe, 2021 71: 102422). Interestingly, both strains appear virulent in the mouse suggesting that the switch orientation *in vitro* may not be linked to virulence *in vivo*.

To acknowledge these points without impacting on the overall narrative of the paper, an additional supplementary figure (Fig. S7) has now been included, which is described in the discussion. This acknowledges the relevance and importance of phase variation, while highlighting that we do not believe it is linked to the phenotypes observed in this study.

With respect to the reconstruction of the mutations in an unaltered background, sadly this is not possible as all attempts to manipulate the *slpA* loci within any wild type *C. difficile* strain, by ourselves and several other groups, have failed; perhaps due to the high constitutive transcription of this gene. While complementation of FM2.5 with all 13 known SlpA types from a plasmid has been previously performed, the maximum amount of protein generated and exported is only equivalent to at most 15-20% of the amount of protein required for an intact S-layer. Whilst this has been sufficient to allow studies of phage sensitivity, it would not provide an opportunity to study the impact of these variants *in vivo*. Further, attempts to increase expression of SlpA from these plasmids has resulted in plasmid instability.

2. Sequencing experiments performed on spores vs 24, 48 etc. hpi convincingly show that the revertants are present in the spore preps. It would be helpful to compare this to the hamster experiments as well where there were no revertants recovered.

Unfortunately, we have no material remaining from the previous work performed using hamsters, and the group no longer have the appropriate Home Office UK licence in place to allow repetition of the study. Analysis of individual colonies recovered from hamsters did show the animals to be infected with FM2.5. by amplification, and sequencing of *slpA* and the use of Multi Locus Variable Analysis (MVLA) for strain identification from individual colonies, but the lack of a larger scale analysis makes it impossible to categorically state that no variants were present within the recovered population in those experiments.

**Part III – Minor Issues: Editorial and Data Presentation Modifications**
Please use this section for editorial suggestions as well as relatively minor modifications of existing data that would enhance clarity.

 Reviewer #1: 1) For the suppressor strains, was the whole genome re-sequenced to ensure that these are true suppressors alleles and not a contaminating strain? There are reports that mice can come pre-colonized with a contaminating C. difficile strain. I suspect these are true suppressor isolates, but for clarity, sequencing them may be useful and has become very inexpensive.

We thank the reviewer for their comment. The recovered strains have been sequenced and analysed. We provide additional data as a supplementary figure (Fig. S2) which shows a phylogenetic tree generated from whole genome analysis of these strains. This reveals that FM2.5_varA_ and FM2.5_varB_ are closely related to FM2.5 and R20291 (showing less than 8 unique polymorphisms compared to FM2.5; in addition to the original point mutation found in *slp*A of FM2.5, which confirms these strains were derived from this strain), indicating their recovery is not a result of pre-colonisation of the mice with an unrelated strain. In addition, all mice used in these experiments were pre-screened for the carriage of *C. difficile* by plating of faeces on ChromID selective plates before challenge with *C. difficile* spores.

2) The importance of the RdeltaD2 S-layer needs expanded upon in the crystallographic section. It is unclear the importance of this strain and why comparisons are being made to it.

The structure of RΔD2 is used as a model for comparison as the only available SlpA structure from R20291, even though it is incomplete (lacks the most external, flexible domain in SLP_L_, domain D2). It is clear from our determined structural models from different S-layer Cassette Types (SLCTs) that different SlpAs can adapt slightly different conformations, particularly in SLP_L_, where the mutations are found. Therefore, to use an experimental model as close to the R20291 structure as possible, we use SlpA RΔD2.

We have expanded on this reasoning within the text, as requested by the reviewer.

3) Figure 3 needs better labeling. Please indicate which alleles each panel represent. For example, 3A should be labeled RvB.

As requested, we have added extra labels to indicate which SlpA the representations refer to throughout the figure.

4) Line 450. It would be helpful to expand upon the differences between mice and hamsters for LL37, etc. and how these differences may / may not lead to suppressor phenotypes.

On re-reading the literature, we believe that we have insufficient evidence to support the statement made about difference in mice and hamster antimicrobial peptides. This has now been removed from the discussion.

5) Lines 478 - 488. It is stated that >2.5% of the spores in the preparations have variations in the slpA gene. Later in the same section, the authors question why animals infected with FM2.5 do not have disease and then go on to propose reasons. One reason that is not discussed is that if the authors are giving 10^5 spores as a dose to the mice, 2.5% = 250 spores with suppressor alleles. It is likely that this low dose of 'actually infectious' C. difficile takes much longer to grow in vivo (between 8 and 9 doublings to get to the original infectious dose).

The text has been modified to strengthen our acknowledgement that the lower rates of recovery at 24 hpi could suggest that the delayed and reduced weight loss may reflect that a lower dose of potentially infectious *C. difficile* may take longer to grow. However, the point being addressed in the discussion is that when these variants do multiply to equivalent numbers, producing similar amounts of toxin, the animals do not become as sick as those infected with the fully virulent R20291.

6) Line 580. Were male or female mice used?

Female mice were used throughout the work. This information is included in the figure legends and has additionally been added to the methods section.

Reviewer #2:

Ln 47-49: The intent of these comparisons and the meaning of this statement is not clear.

The entire abstract has been re-written to enhance clarity throughout. The section indicated here has been re-written to clarify which comparisons were undertaken and the conclusions that can be drawn from the data presented.

Ln 146: This 2020 reference for the long-established correlation of weight loss and disease severity is not the first nor the most relevant reference to support the statement.

The authors completely concur with this comment. This reference was chosen as most early references use the VP10463 as the infecting strain rather than R20291 (UK1) or use cefoperazone as the antibiotic of choice to suppress the microflora. All show the overall same pattern of acute disease with peak weight loss at around 48 h post infection. However, in line with the reviewer’s comment, several other earlier references have now been added in support of this statement.

Reference to Fig 4C appears following the reference to Fig 1. Figures should be referenced in the order that they appear in the manuscript.

This has now been removed from the text, as requested.

Fig 1 (SlpA deficient C. difficile is less pathogenic in a murine model of infection). The legend mentions statistical analyses and p values, but none are shown in the figure. Were statistical denotations left out unintentionally or were the results not significant? If not, the figure title should be amended.

As mentioned above, the figure uploaded lacked the relevant information regarding statistical significance. We apologise for this unintentional error, and have now included a much more detailed analysis, as described above.

Ln 207-209 mentions that the revertants RvA and RvB were the most common revertants isolated. How many revertants were identified? What other reversions occurred? Were there compensatory mutations outside of the slpA locus? How many independent clones were tested and sequenced?

We appreciate the reviewer’s comment and agree that the terminology of ‘most common revertant’ is not entirely reflective of the way these variants were initially identified. FM2.5_varA_ and FM2.5_varB_ were the first and second variants identified during a study of FM2.5 infection in mice, performed to compare with our previous experiments in hamsters. Modifications in *slpA* were initially identified following isolation of individual clones, followed by PCR amplification and sequencing of the region around the original mutation site. At least 20 individual clones, randomly selected from larger than expected colonies, were tested per experiment. In each animal experiment, in which different batches of spores were used, recovery of colonies from faecal pellets over at least 5-days, identified at least one variant; FM2.5_varA_ (experiment 1), FM2.5_varB_ (experiment 2), FM2.5_varC_ (experiment 3) and FM2.5_varD_ (experiment 4). In addition, FM2.5_varA_ and FM2.5_varC_ were observed in more than one experiment. Due to the methodology used, it is difficult to know whether other clones existed in low numbers that were not identified. The wording used to describe the identification of variants has been changed for clarity.

Fig 2 legend (ln 229): The authors should clarify in the figure and legend that C shows truncated products for all variants.

Modification of the *slpA* sequence in the variants results in the restoration of the reading frame of the gene, thus preventing premature translational termination observed with FM2.5. This means the full *slpA* gene, although slightly modified for the variants, is expressed in its entirety and the gene product is not truncated. The figure has been modified to show a similar portion of the *slpA* gene beyond the premature stop codon in FM2.5 and this difference has been described for clarity in the figure legend.

Discussion ln 449-452: there is little known to support this conclusion. It is likely there are many factors both known and unknown that contribute to the difference in pathogenesis in the different animal models of CDI.

The authors completely agree with this statement but were only attempting to offer possible explanations for this observation. This section has now been removed.

Fig S3: the colors representing different strains are inconsistent between the graphs.

Apologies, this was an oversight that has been rectified.

Fig S3: Bacterial cells are resistant to killing in feces. If all cells were plated on medium that could support vegetative and spore cell growth, how do the authors know that the resulting CFU were derived from spores or vegetative cells? What controls were performed to ensure that vegetative cells were killed and only spores remained?

There was an error in the description within the legend, which has now been corrected. This should have reported the counts to reflect the total number of organisms in the population (spores and vegetative cells) and the spores alone (as is described in Fig. 1). The methodology, used by multiple research groups, involves the treatment of samples for 20 min at 65^o^C, which kills the vegetative cells but does not impact on the capacity of the spores to germinate. A suitable reference has been offered in support of this approach.

Reviewer #3: 1. Is it possible to run alphafold on the RvA mutant?

We initially used Alphafold 2 to make predictions of all SlpA sequences: R20291 (as we only have a partial model, lacking the most external domain, D2), variant A and variant B. However, the predicted models always had SLP_L_ oriented not towards the external environment but towards the cell wall, which contradicts experimental data (Willing et al., 2015). Therefore, we tried to crystallise all forms, but were only able to obtain an experimental model for variant B. The striking differences between the AF2 prediction and the experimental model for SlpA_varB_ further supports our choice of not using those predictions in our analysis and comparisons. Conversely, SWISS-MODEL predictions, even in the absence of a template, preserved the known relative orientation between SLP_L_ and SLP_H_ and are used as a comparison in our manuscript.

We have added a few sentences to reflect this in the manuscript.

2. Does repeated sporulation/germination cycles in vitro produce increased recovery of revertants?

Sequential sporulation and germination cycles for a least 4 cycles for FM2.5 have been performed, with no evidence of variant selection *in vitro*. However, this observation was based solely on the absence of the larger colony size detected following plating of in vitro grown cultures and not by sequencing of selected clones. It is possible that this will occur, but selective pressure *in vitro* is possibly low and it may take months before such a phenotype becomes apparent. In addition, this strain has been routinely propagated since its first identification in 2016 by us and other groups, with no modifications noted, suggesting that such conditions do not offer any selective advantage for those clones in which SlpA has been restored.

3. Can you discuss the number of times C. difficile doubles in 24hrs in mouse does that differ with a hamster?

This is difficult to compare directly as the dose of spores used to reproducibly infect hamsters is 10-100-fold less than that required to infect mice (Buckley AM, Spencer J, Candlish D, Irvine JJ, Douce GR. Infection of hamsters with the UK *Clostridium difficile* ribotype 027 outbreak strain R20291. J Med Microbiol. 2011;60:1174-1180). In both species, the organism duplicates through at least 4-5 generations, with a slightly higher rate observed in hamsters.

4. Likely not in the scope of this but what is the phenotype of RvA or RvC in hamster model of infection.

As highlighted above, the group no longer holds the appropriate licencing for these experiments, so sadly this is not feasible.
