# Supplementary material for: An intact S-layer is advantageous to Clostridioides difficile within the host
Source: PLoS Pathog. 2023 Jun 29;19(6):e1011015. doi: 10.1371/journal.ppat.1011015 (PMC10310040; doi:10.1371/journal.ppat.1011015)
Supplement: Attachment — Submitted filename: Final responses to referees.docx [file ppat.1011015.s012.docx]

**Response to Reviewers**

There was some Reviewer disappointment that the mutations could not be rebuilt in the parent strain, leading to some comments that the current data is being over interpreted. In general the manuscript does indicate that the findings support or suggest, rather than prove, the importance of the S-layer but this should be the uniform message in the manuscript, e.g., the title is perhaps too definitive regarding the findings.

**We understand and share the frustration with respect to re-capitulation of these genetic changes within the parental strain but hope that, as experts in the field, the reviewers share a clear understanding of the complexities associated with genetic manipulation of *C. difficile* in general, without the additional complications associated with this highly regulated system. Due to the nature of the changes and the difficulties associated with manipulation of the *slpA* gene, it is not technically possible to recreate these variants.**

**We have shown in multiple experiments that there is a strong selective pressure for restoration of the S-layer *in vivo* (see figures 1 and 4). The title, as given, summarises our work and highlights the key observation that having an intact S-layer is an advantage within the host, as described and discussed throughout the manuscript.**

**Major Changes:**

Reviewer #1: The authors have adequately addressed my prior concerns. That said, the terms 'revertants' still appear in Figure 2, 4 and should be fixed.

**We apologise for missing these terms in our original edits. These have now been modified in line with our description of these strains as variants.**

Reviewer #2: The differences in the ratio of variants A and B in their respective input populations for animal studies is not provided (variant C ratio is provided in Fig 2g, but this variant was not shown or tested). The authors state that the ratio was “low” for the other variants, but no data are provided to suggest the starting ratios for variant A or B. Without this information, few conclusions can be drawn from the in vivo data.

**There is no data provided for the relative proportions of variants A and B within the respective inoculum, as the focus of the initial experiments was to establish whether FM2.5 showed attenuation in the mouse model of disease. As described in the text, the recovery of variants was a serendipitous outcome, which we have investigated in more detail by performing the studies described in this work. In fact, the experiment in which variant C was identified, was specifically designed to address the question of whether the variants were present in the initial inoculum, or whether they arose later within the host. These modifications are stochastic events, confirmed by the detection of more than one variant in different experiments. Within the specific spore preparation used in the experiment involving amplicon amplification and sequencing to identify variants (Fig. 2G), only variant C was identified above the detection limit. We cannot exclude the possibility that variants A, B or D or others were present in this preparation but at a low frequency, which was beyond the discretionary power of the sequencing data. Based on our overall observations, each spore preparation is likely to contain low numbers of different variants, clones of which outgrow and outcompete the mutant within the population *in vivo*, supporting our observation that having an intact S-layer is advantageous in this environment.**

The histology studies are underpowered and insufficient to draw conclusions. Fig 1e) How can PBS show high positive histology scores (appears to only have two animals)?

**These figures (Fig. 1e and Fig. S1c) have now been modified to include additional data points and scores for each of the three sections from four different animals that were sampled. To avoid confusion regarding the scoring of 5 for the PBS control tissue, we have adjusted our scoring to use 0 for no change, 1 for slight change, 2 for moderate change and 3 for significant change, giving a new total of 15 points if all 5 features are significantly altered. We have modified the figure legend and the description in the materials and methods to improve clarity.**

Full RNA-seq expression analyses should be provided. How was slpA expressed in the different strains and what fraction of the transcript was represented in the data sets?

**Tables of differentially expressed genes identified in comparative analysis of all strains are now provided in the form of an Excel spreadsheet within the supplementary information.**

**In our transcriptomic analysis, *slp*A was the most highly expressed transcript in all of our strains. This is not surprising given that the S-layer is largely comprised of approximately 600,000 copies of SlpA. In FM2.5, the *slpA* gene is transcribed as normal but not translated as a full length protein due to the frame-shift mutation early in the ORF. Consequently, as the transcripts are generated to equivalent levels by all our strains, *slpA* does not appear in our list of differentially regulated genes. A number of sentences have been added to the text to highlight the fact that the *slpA* transcript is the most highly expressed transcript in all strains.**

The limited data that are shown in Figure 6 appear biased towards sporulation and virulence, but there are many differentially regulated transcripts. The mutant has growth defects, which likely account for much of the differences in gene expression.

**As the focus of this work was on to establish how *slpA* variants may affect virulence, analysis represented in Figure 6 highlights differentially expressed genes with known links to disease within the animal. Although FM2.5 does have growth defects *in vitro*, as we have previously described (Kirk *et al*., 2017), these appear linked to the modification of S-layer expression.**

**We have provided the full comparative data for all differentially expressed genes between these strains in an Excel spread sheet making the full data accessible to the community. We welcome any contributions that would further our understanding of the role of the S-layer in *C. difficile* disease that this resource might provide.**

Whole-genome sequence data is not available in a repository.

**We would like to apologise for our oversight upon resubmission. These data have been submitted to a repository and are available at** <https://www.ncbi.nlm.nih.gov/geo/query/acc.cgi?acc=GSE205747>.

**Minor changes:**

Reviewer #2: The mutations that arise in FM2.5 to restore SlpA function are compensatory, rather than revertants, since they do not revert to the original sequence.

Considering that the authors have been unable to delete the slpA gene, it seems likely that the FM2.5 mutant is a truncation with low function, rather than a true null (non-functional) mutant.

**The location of the stop codon in the *slpA* gene results in the potential expression of a truncated version of the protein, corresponding to the signal peptide, and 73 amino acids of the mature low molecular weight S-layer protein (SLP_L_). This peptide corresponds to a small fragment of domain D1 containing two α-helices and an incomplete β-sheet (β1-α1-β2-β3-α2, see SI Fig. 1 in Lanzoni-Mangutchi *et al*., 2022) of SLP_L_ protein. This sequence does not include the cell wall binding domains in SLP_H_, or the low molecular weight interacting domain (LID). We have shown that both these domains are essential for S-layer structural stability and for maintaining the SlpA proteins anchored to the cell wall (Lanzoni-Mangutchi, *et al*., 2022; Wiling *et al*., 2015). This means that even if the protein is expressed and successfully secreted, there is no mechanism by which it can attach to the cell surface.**

**In addition, highly specific polyclonal antibodies to SLP_L_ have repeatedly failed to detect protein on either the surface of the organism or from the spent medium from bacterial cultures indicating that if this severely truncated protein is expressed it is unlikely to be secreted from the cell.**

**Taken together, these observations suggest that any peptide expressed would not be functional as it wouldn’t be present in the S-layer or anchored to the cell wall. FM2.5 is therefore a true null, non-functional *slpA* mutant.**

Ln 153. “..strain FM2.5 showed consistently and significantly less weight loss”. This statement is not supported by the data presented in Fig 1a. The FM2.5 mutant resulted in statistically greater weight loss at the 96 hour timepoint.

**The data in Fig. 1a shows that, in comparison to the WT strain, mice infected with FM2.5 showed significantly less weight loss at 24 and 48 hpi, as described and discussed in the text, with a mean reduction in weight loss of only 6% at 48 hpi. The weight loss of these animals was not significantly different from those animals treated with PBS. By comparison, R20291-infected animals, lost 10% at 24 hpi and nearly 15% at 48 hpi.**

**At 96 h, while mice infected with R20291 had fully recovered their pre-infection weight and showed no statistical difference to PBS treated animals, FM2.5 infected mice showed a statistically significant weight loss when compared to the uninfected controls. The figure legend and the relevant sections of the text have been modified to clarify this point.**

Ln 154. “measurement of total C. difficile in faecal material showed comparable levels of total numbers of bacteria shedding at 24 and 72 hpi (Fig. 1b), the recovered number of spores was significantly lower in animals infected with FM2.5 at 24 h.” The authors do not demonstrate that they measured spore viability rather than vegetative cell susceptibility to heat. Controls are needed to support this conclusion.

**Use of heat treatment (65 ^0^C for 20 min) is a well-established method for differentiation of spores from total viable counts in *C. difficile* populations. Previously published data (supplementary Fig. S5; Kirk *et al.*, 2017, and included here for clarity) has shown that FM2.5 spores subject to this treatment are viable and are as capable of germination as untreated spores. In this previous study, we also demonstrated that direct visual counting of spores and vegetative cells for R20291 and FM2.5 were consistent with enumeration with and without heat treatment (supplementary Fig.****S7; Kirk *et al*., 2017).**

**It is not clear what additional controls would be required and given the data available it would appear ethically unjustified to cause further animal suffering, when *in vitro* data is available that supports our analysis.**

Ln 157. There is not sufficient data presented to support the conclusion that the mutant sporulated less efficiently in vivo.

**It is unclear what additional information is required here, as at 24 hpi, fewer spores were recovered from the faeces (Fig. 1b) and tissue (Fig. 1c) of infected animals despite equivalent total viable counts. Data shown above and generated at later time points suggests that FM2.5 spores are viable and can germinate normally. While some of the spores recovered could be attributed to variant amplification, many colonies were positively identified as having the FM2.5 sequence from heat treated plates, indicating that these spores are capable of germination under these conditions. Thus, their absence at early time points in the infection, linked to the transcriptional data which revealed sporulation to be affected in this strain, would support our interpretation that FM2.5 sporulates less efficiently *in vivo*.**

Have the authors tested if the S-layer provides protection against the selective ChromID agar components? Because the FM2.5 mutant is highly susceptible to surface insults, the ChromID selection may bias against the FM2.5 mutant.

**ChromID agar is a patented product produced by BioMerieux, therefore it is difficult to know the impact of specific components. As shown in Fig. 2a, both small FM2.5 colony morphology and larger, variant morphology are detectable in ChromID plates. Moreover, we have previously performed comparative bacterial counts (cfu/ml) of cultures of R20291 and FM2.5 (total and spore counts) on ChromID in comparison to CCFA agar, with no suggestion of bias against the recovery of FM2.5.**

Ln 452-454. Have the authors considered that the differences in gene expression in the FM2.5 mutant are due to its growth defect, rather than a specific differential expression of toxin and sporulation genes?

**From all the data we have been able to generate thus far, the growth defects observed appear to be linked to the loss of the S-layer, and consequently the two are intrinsically linked. The focus of this paper is not to provide an explanation for the mechanism by which the S-layer modifies toxin and sporulation gene expression, but rather to provide evidence that recovery of an intact S-layer offers a significant advantage to the bacterium within the host.**
