# Supplementary material for: An intact S-layer is advantageous to Clostridioides difficile within the host
Source: PLoS Pathog. 2023 Jun 29;19(6):e1011015. doi: 10.1371/journal.ppat.1011015 (PMC10310040; doi:10.1371/journal.ppat.1011015)
Supplement: Attachment — Submitted filename: response to reviewers.docx [file ppat.1011015.s013.docx]

Response to Reviewers

A previous reviewer asked about the presence of variants A and B in the input population for the animal studies. The response to Reviewer Comments of Revision 1 indicates that the presence of variants A and B in the input were below the detection limit. Please clearly state this in the Results.

As requested, and in response to reviewer comments, the manuscript has now been modified to provide further clarity about the failure to detect variants A and B in the spore (input) preparation or the faecal samples from the unbiased amplification of the *slpA* amplicon analysis that was undertaken (Lines 272 -278 of the annotated modified manuscript).

The same reviewer also questioned the power of the histology analysis and the Response to Reviewer Comments indicates that, in response, more microscope sections were examined. However, line 206 of the Figure 1 legend indicates that the histologic scoring for Fig. 1e includes the caecum and upper and lower colon sections (combined) after challenge. This "lumping" is inappropriate. Please present this histopathology data separately for each tissue site rather than in combination. Should the results not be significant, they can be described as a non-significant trend.

Regarding histology scoring, we have removed the errant description in the legend of Figure 1 and wish to reassure the editorial team that there has been no ‘lumping’ of tissue scoring. The data in Figure 1e represents the scoring of caecal tissue alone, with statistical comparison only performed between treatments on the same tissue. Similarly, data represented in Fig S1c, shows data from scored tissue from either the upper or lower colon, with statistical analysis performed only between treatment groups from the same tissue and not between different regions within the colon. These different regions (within the same tissue) are highlighted in the text below the figure.

Please revise Figure 1e (and supplemental Fig 1) and the Results text accordingly. Last, you are encouraged to deposit the RNA-Seq data in a repository.

Finally, all the raw RNA data has been deposited in the GEO repository <https://www.ncbi.nlm.nih.gov/geo/query/acc.cgi?acc=GSE205747>. Currently this is password protected (**obczowsshpilhsl)** for reviewing purposes. This data will be released on acceptance of the manuscript. The annotated list of differentially expressed genes identified by our analysis has been made available as an excel spreadsheet (Spreadsheet S1) which has been provided as supplementary information. Al figures uploaded have been subject to PACE digital diagnostic tool.
